# Supplementary material for: Robustness of a multivariate composite score when evaluating distress of animal models for gastrointestinal diseases
Source: Sci Rep. 2023 Feb 14;13:2605. doi: 10.1038/s41598-023-29623-8 (PMC9929045; doi:10.1038/s41598-023-29623-8)
Supplement: Supplementary file 1 — Supplementary Information. [file 41598_2023_29623_MOESM1_ESM.docx]

**Supplemental Material**

**Supplementary Figure S1**


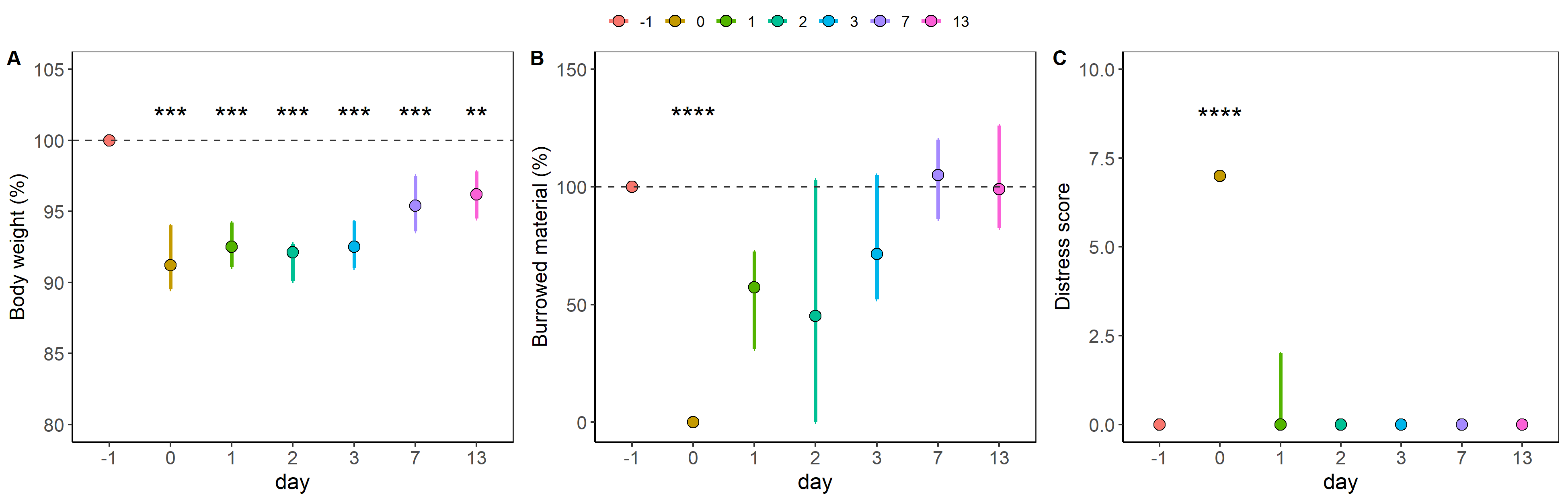


**Supplementary Figure S2**

**
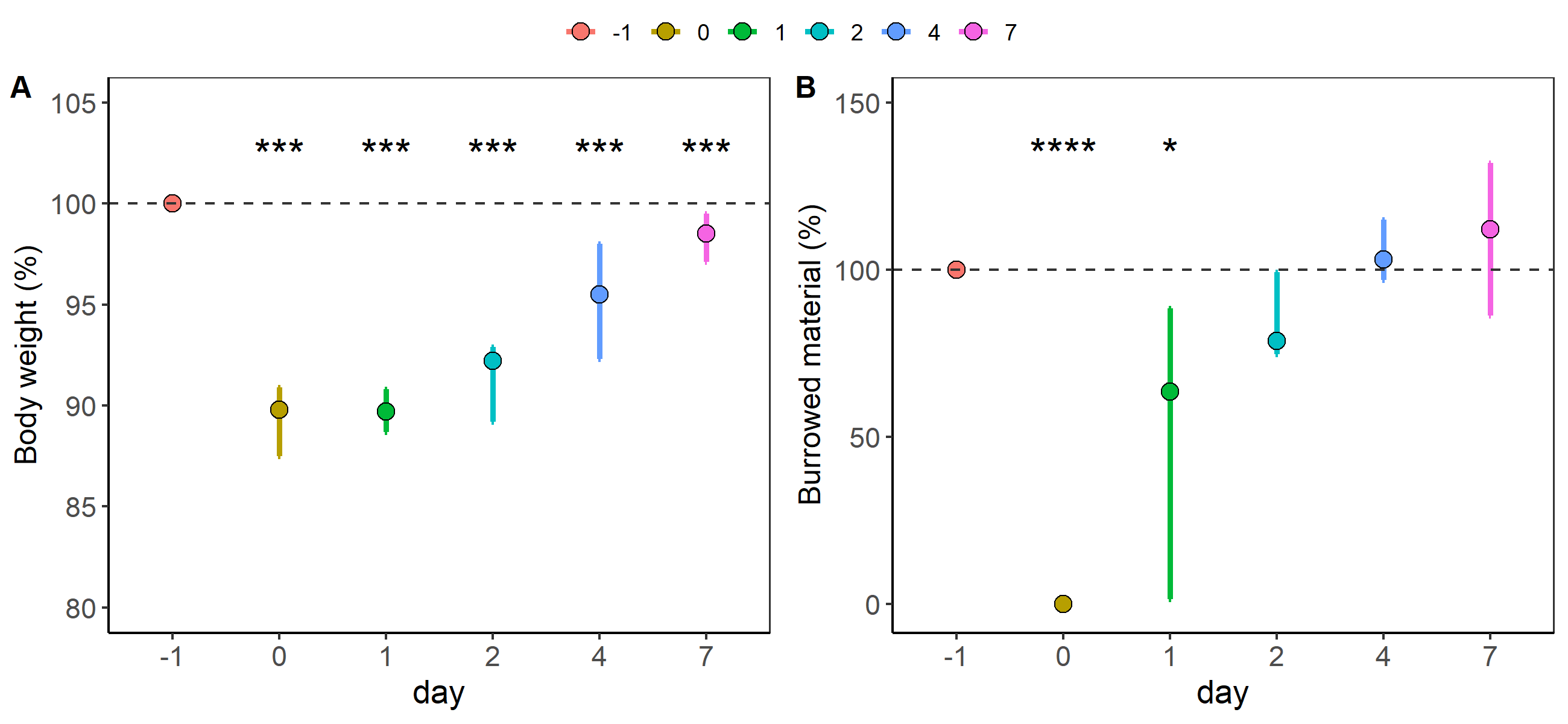
**

**Supplementary Tables**

The following tables (T1-T4) provide the p-values of the non-parametric Dunn’s test for all pairwise comparisons of the experimental days vs baseline values (day -1). The significant (Holm-corrected) *within*-subgroup comparisons are reported in the results as adjusted p-values (p_adj_).

**Table T1 - Pancreatic Cancer**

**Table T2 – Pancreatitis**

**Table T3 – CCL_4_**

**Table T4 – Bile Duct Ligation**
