# Supplementary material for: Robustness of a multivariate composite score when evaluating distress of animal models for gastrointestinal diseases
Source: Sci Rep. 2023 Feb 14;13:2605. doi: 10.1038/s41598-023-29623-8 (PMC9929045; doi:10.1038/s41598-023-29623-8)
Supplement: Supplementary file 2 — Supplementary Legends. [file 41598_2023_29623_MOESM2_ESM.docx]

**Supplementary Fig. 1:** Description of the reference set (implantation of a telemetric transmitter in laboratory A). The percentage of body weight (A), the percentage of burrowing activity (B), and a distress score (C) were evaluated on the indicated days. (A) The daily data showed normally distributed estimators after the bootstrapping in the percent body weight variable. Therefore, a *repeated-measures* ANOVA was used to determine differences between experimental days. The percent body weight variable showed significant differences over time (F(6,54)=56.562, p<0.0001, η_G_^2^=0.672). Dunnett’s post hoc test was used to determine significant differences on the indicated days compared to day -1 (**p_adj_≤0.01, ***p_adj_≤0.001, ****p_adj_≤0.0001). (B) The burrowing data did not show normal distribution on the experimental days. Therefore, a Friedman test with the day as the *within-subjects* variable was performed to assess the development over time. The burrowing variable showed significant differences over time (χ^2^=33.9, df=6, p<0.0001). Dunn’s post-hoc test with Holm’s correction revealed that there were significant differences between day -1 (baseline) and day 0 (p_adj_<0.0001). (C) The Friedman test of the distress score showed a significant difference between days as the *within-subjects* variable (χ^2^=48.6, df=6, p<0.0001). Dunn’s post-hoc test with Holm’s correction shows a significant difference between day -1 (control) and day 0 (p_adj_<0.0001). The graphs depict the bootstrapped median estimator on each experimental day and the 95 % confidence intervals. A-C: n=10.

**Supplementary Fig. 2:** Description of the validation reference set (implantation of a telemetric transmitter in laboratory B). The percentage of body weight (A) and the percentage of burrowed material (B) were evaluated on the indicated days. (A) The daily data showed normally distributed estimators after the bootstrapping in the percent body weight variable. Therefore, a *repeated-measures* ANOVA was calculated to determine differences between experimental days. The percent body weight variable showed significant differences over time (F(5,60)=82.01, p<0.0001, η_G_^2^=0.746). Dunnett’s post-hoc test was used to determine significant differences (***p_adj_≤0.001) at the indicated days compared to baseline (day -1). (B) The burrowing data did not show normal distribution on the experimental days. Therefore, a Friedman test with the day as the *within*-subjects variable was performed to assess the development over time. The burrowing variable showed significant differences over time (χ^2^=43.874, df=5, p<0.0001). Dunn’s post-hoc test with Holm’s correction revealed that there was a significant difference between day -1 and day 0 (****p_adj_<0.0001) as well as between days -1 and 1 (*p_adj_=0.026). The graphs depict the bootstrapped median estimator on each experimental day and the 95 % confidence intervals. A-B: n=13.
